# KIAA0101 is associated with human renal cell carcinoma proliferation and migration induced by erythropoietin

**DOI:** 10.18632/oncotarget.5876

**Published:** 2015-11-12

**Authors:** Shengjun Fan, Xin Li, Lu Tie, Yan Pan, Xuejun Li

**Affiliations:** ^1^ State Key Laboratory of Natural and Biomimetic Drugs, Department of Pharmacology, School of Basic Medical Sciences, Peking University Health Science Center and Beijing Key Laboratory of Tumor Systems Biology, Peking University, Beijing, China

**Keywords:** EPO, KIAA0101, renal cell carcinoma, proliferation and migration, proteomics

## Abstract

Erythropoietin (EPO) is a frequently prescribed anti-anemic drug for patients with advanced renal carcinoma. However, recent evidence from clinical studies suggested that EPO accelerated tumor progression and jeopardized the 5-year survival. Herein, we show, starting from the *in silico* microarray bioinformatics analysis, that activation of Erythropoietin signaling pathway enhanced renal clear carcinoma (RCC) progression. EPO accelerated the proliferative and migratory ability in 786-O and Caki-2 cells. Moreover, comparative proteomics expression profiling suggested that exogenous EPO stimulated RCC progression *via* up-regulation of KIAA0101 expression. Loss of KIAA0101 impeded the undesirable propensity of EPO in RCC. Finally, low expression of *KIAA0101* was associated with the excellent prognosis and prognosticated a higher 5-year survival in human patients with renal carcinoma. Overall, KIAA0101 appears to be a key promoter of RCC malignancy induced by EPO, which provide mechanistic insights into KIAA0101 functions, and pave the road to develop new therapeutics for treatment of cancer-related and chemotherapy-induced anemia in patients with RCC.

## INTRODUCTION

Renal cell carcinoma (RCC) constitutes about 2 to 3% of all adult malignancies with approximately 271, 000 new cases and 116,000 related deaths estimated worldwide [1]. RCC is classified histologically into several types, including clear cell RCC (ccRCC), the most common and aggressive type accounting for 70 to 80% of all kidney cancers [2]. By far, multiple etiological factors including obesity [3], dietary habits [4], alcohol drinking [5], smoking [6] and even occupational exposure to specific carcinogens [7] have been attested to have a first-degree relative with RCC malignancy. Besides, genetic mutations or functional loss of tumor suppressor genes such as *VHL*, *BAP1*, *PBRM1* and *SETD2*, also contribute to RCC cachexia [8]. Interestingly, in some cases, malignancies are often manifested owing to their clinical manifestations including skeletal muscle atrophy, anemia, weakness or anorexia [9]. All of these cancer-associated systemic diseases (CASS) entail a significant reduction in quality of life and even shorten 5-year survival rate [10]. Among them, anemia is a frequent finding in cancer patients occurring in more than 40% of cases [11]. The nature of cancer-induced anemia is multi-factorial with potential contributing factors including blood loss from surgery, tumor-associated bleeding, nutritional deficiencies, chronic inflammation and even the myelosuppressive effects of cytotoxic chemotherapy [11].

Accumulating clinical evidence from large randomized trials indicated erythropoiesis-stimulating agents (ESAs) benefit individuals with cancer-induced anemia. ESAs treatment, especially for recombinant human erythropoietin (r-Hu EPO), has become an indispensable procedure of supportive therapy for cancer patients with anemia [12, 13]. EPO elevates red blood cell count, hemoglobin and hematocrit peripheral blood system, which results in the improvement of quality of life in patients with anemia. However, a double-edged sword acting mediated by EPO in cancer patients gradually embodied and its beneficial effects on cancer patients has been recently challenged. Large clinical researches aimed at the correction of breast cancer [14], lung cancer and cervical cancer [15] have raised the question that EPO treatment jeopardized the overall survival rate in cancer patients with anemia. Although conflicting data on EPO have been implicated to play a role in tumorigenesis and progression, little is known about whether and how EPO regulate RCC progression.

KIAA0101, also referred as PAF15 (proliferating cell unclear antigen-associated factor), encodes a PCNA (proliferating cell nuclear antigen)-associated factor [16]. KIAA0101 has been verified to regulate DNA synthesis and cell cycle progression [17]. Inhibition of KIAA0101 in cancer cells improved cancer cells sensitive to ultraviolet. In addition, levels of KIAA0101 are also found to be up-regulated in cancer tissues from patients with esophageal, gastric and lung carcinoma [18–20]. Hence, KIAA0101 potentially functions as an oncogene in tumor progression. However, it is still unknown whether EPO regulates KIAA0101 expressed in RCC of clear cell type. It is a clinically important question in light of the reported unwanted side effects by EPO in tumor patients with anemia. Herein, we demonstrated KIAA0101 as a positive, yet previously unrecognized, regulator of ccRCC proliferation and migration induced by recombinant human erythropoietin (r-Hu EPO). Our work suggests an unfavorable role of KIAA0101 in the mechanism by which EPO elicits an undesired response that maybe important to sustain RCC progression.

## RESULTS

### CcRCC regulatory network modules construction

**Figure 1:**
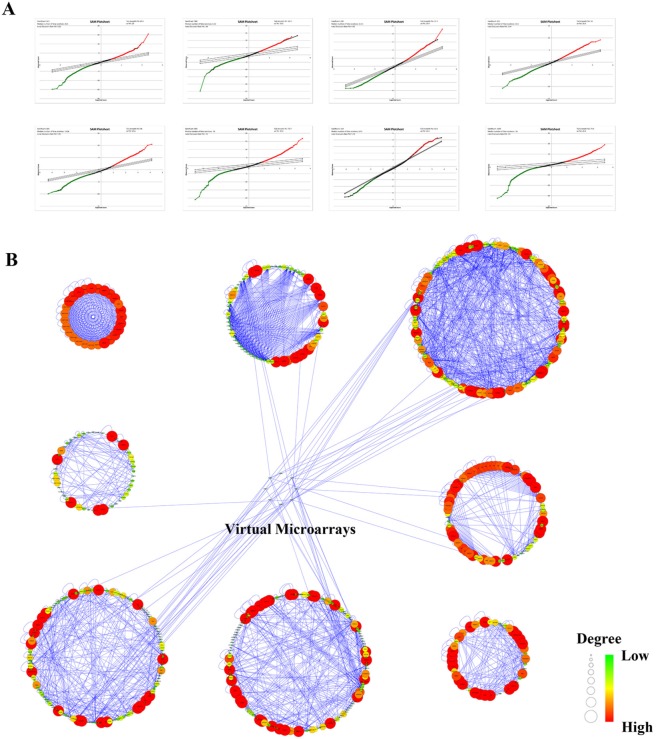
Bioinformatics analysis of human renal cell carcinoma microarray originated from the public available GEO and ArrayExpress warehouses **A.** SAM plot sheets of the stimulated microarray datasets originated from the integrated cohort of microarray downloaded from GEO and ArrayExpress. In the plot, red dots represent genes that were up-regulated, while green dots stand for down-regulated genes. **B.** Signaling networks regulated by RCC from the manually curated human cancer signaling atlas.

**Figure 2:**
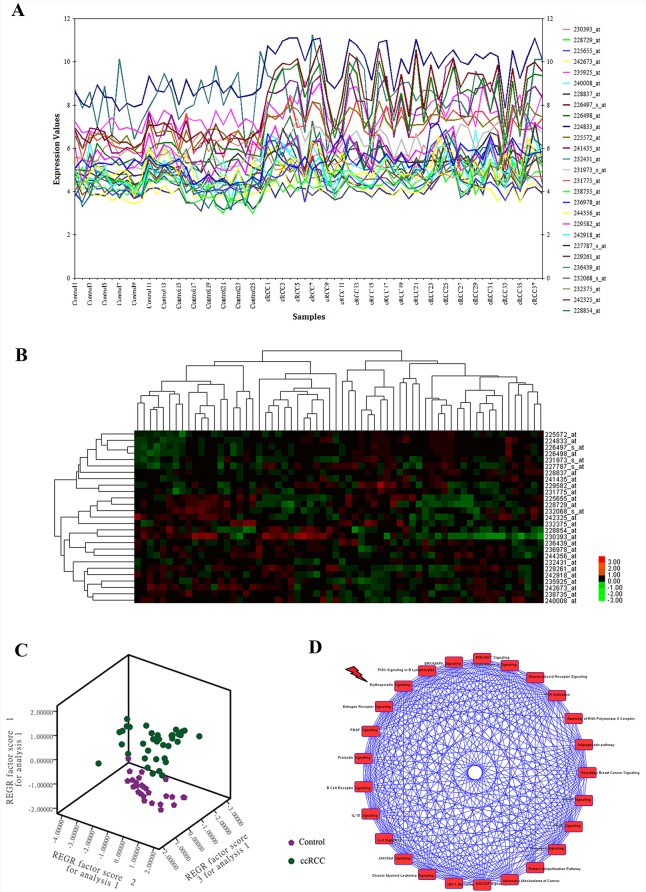
CcRCC gene signatures identification and signaling pathway enrichment analysis of ccRCC regulatory networks **A.** Expression plot, **B.** *K*-means hierarchical clustering and **C.** principal component analysis (PCA) analyses of gene signatures driven the renal carcinoma regulatory networks. **D.** Global canonical pathway enrichment analysis results basing on the Ingenuity Pathway Analysis (IPA) platform.

Totally, 22,639 probes were matched in the integrated cohort from three different ccRCC microarrays (Supplementary material 1). To elevate the reproducibility and obtain stable gene signatures driven ccRCC progression, virtual microarray datasets originated from the combined metadata were generated randomly (Table 1) and performed significance analysis of microarray (SAM) analysis respectively using student's *t* test comparison, which resulted in the generation of a cohort of up (red dots) and/or down (green dots) regulated genes (Figure 1A). To understand how these signatures regulate ccRCC progression, we matched the relationships between signatures and human cancer signaling atlas, which were obtained from literature curation and established warehouses. As shown in Figure 1B, 6 out of 8 network modules derived by the 8 simulated microarrays were thought to be involved in ccRCC and 27 gene signatures have been attested to be robust according to the gene expression (Figure 2A) and unsupervised hierarchical clustering analysis (Figure 2B). In addition, three dimension principal component analysis (PCA) also indicated that 100% (27/27) of ccRCC patients could be correctly classified from the vehicle groups (Figure 2C). To further understand the biological processes involved in the pathogenesis, we performed a pathway enrichment analysis in terms of the global canonical pathway using Ingenuity Pathway Analysis (IPA), which represents immunology and inflammation pathways (such as B cell receptor signaling, IL signaling, IGF signaling, GM-CSF signaling pathway, *etc*.), Erythropoietin signaling, ERK/MAPK signaling and PI3K/AKT signaling pathways were closely associated with the human renal carcinoma protein-protein interaction (PPI) network (Figure 2D).

**Table 1:** Randomly simulated microarray datasets from the integrated human renal carcinoma microarray

Rank	Virtual microarray datasets
1	Control(GSM146804, GSM12078, 32GM cortex_04i12879, 36 MMl cortex_04i18916, 40RR cortex_04i20257); Cancer(GSM146808, GSM12274, 31NR tumor_04i12877, 45DM tumor_05i5902, 46SA tumor_05i6348, 47CA tumor_04i3579, 49CA tumor_05i6348)
2	Control(GSM146800, GSM146814, GSM146816, 33BV cortex_04i13776, 35PA cortex_04i18143); Cancer (GSM146803, GSM146805, GSM146811, GSM146813, GSM12083, GSM12299, 28RA tumor_04i3579)
3	Control (GSM146798, GSM11827, GSM12099, GSM12269, 37BA cortex_04i19473); Cancer (GSM146817, GSM12287, GSM12412, 27CG tumor_03i16741, 37BA tumor_04i19473, 50PC tumor_05i9837, 52CA tumor_05i11034)
4	Control (GSM146809, GSM146810, 28RA cortex_04i3579, 44DE cortex_05i3989, 51MI cortex_05i10081); Cancer (GSM146799, GSM146815, GSM11815, GSM12101, GSM12106, GSM12448, 44DE tumor_05i3989)
5	Control (GSM146802, GSM146806, GSM146812, GSM11810, 41SG cortex_04i20655, 50PC cortex_05i9837); Cancer (GSM146801, GSM146807, GSM11832, GSM12069, GSM12301, 32GM tumor_04i12879, 33BV tumor_04i13776, 36 MMl tumor_04i18916, 40RR tumor_04i20257, 51MI tumor_05i10081)
6	Control (GSM146798, GSM146800, GSM146802, GSM146804, GSM146806, GSM146809, GSM146810, GSM146812, GSM146814, GSM146816); Cancer (GSM146799, GSM146801, GSM146803, GSM146805, GSM146807, GSM146808, GSM146811, GSM146813, GSM146815, GSM146817)
7	Control (GSM11810, GSM11827, GSM12078, GSM12099, GSM12269); Cancer (GSM11815, GSM11832, GSM12069, GSM12083, GSM12101, GSM12106, GSM12274, GSM12287, GSM12299, GSM12301, GSM12412, GSM12448)
8	Control (28RA cortex_04i3579, 32GM cortex_04i12879, 33BV cortex_04i13776, 35PA cortex_04i18143, 36 MMl cortex_04i18916, 37BA cortex_04i19473, 40RR cortex_04i20257, 41SG cortex_04i20655, 44DE cortex_05i3989, 50PC cortex_05i9837, 51MI cortex_05i10081); Cancer (27CG tumor_03i16741, 28RA tumor_04i3579, 31NR tumor_04i12877, 32GM tumor_04i12879, 33BV tumor_04i13776, 36 MMl tumor_04i18916, 37BA tumor_04i19473, 40RR tumor_04i20257, 44DE tumor_05i3989, 45DM tumor_05i5902, 46SA tumor_05i6348, 47CA tumor_04i3579, 49CA tumor_05i6348, 50PC tumor_05i9837, 51MI tumor_05i10081, 52CA tumor_05i11034)

### CcRCC cells secrete endogenous EPO and express authentic EPO receptor protein

**Figure 3:**
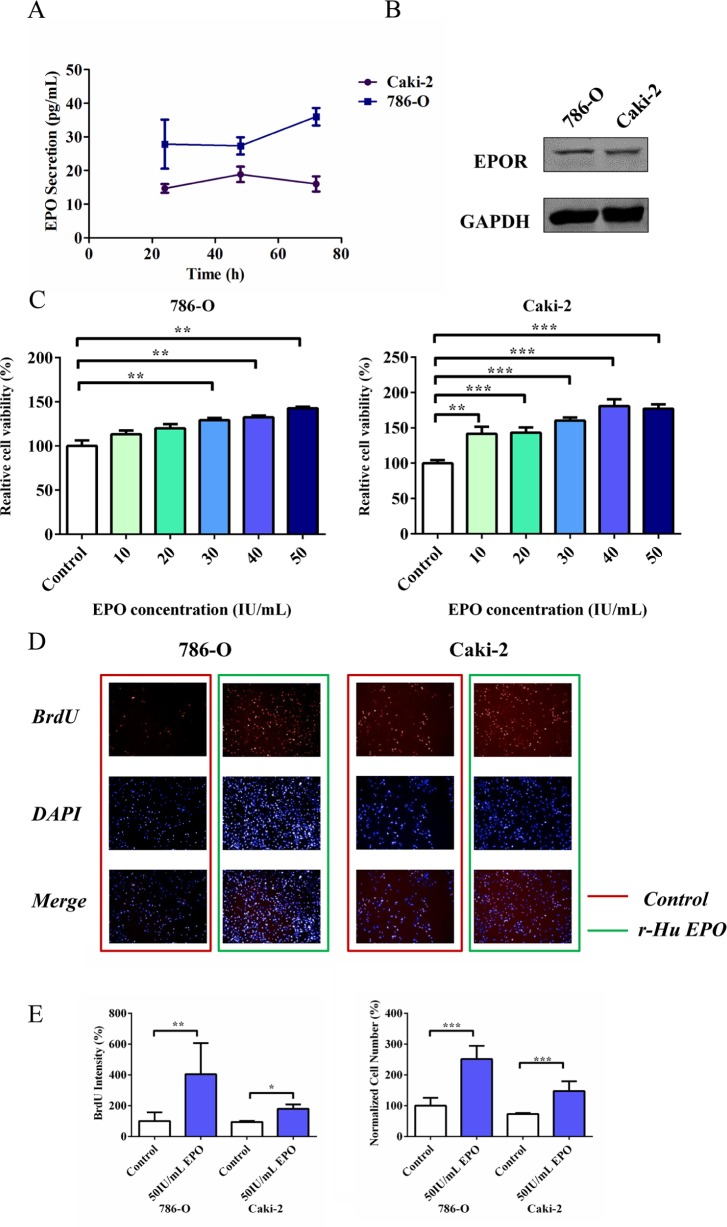
Proliferative and migratory capacity of 786-O and Caki-2 cells induced by exogenous EPO **A.** Endogenous EPO secretion of 786-O and Caki-2 cells determined using ELISA kit. **B.** Western Blot analysis of EPOR expression in 786-O and Caki-2 cells. **C.** MTS assay for cell proliferation in 786-O and Caki-2 cells induced by exogenous EPO. **D.** BrdU proliferation of 786-O and Caki-2 cells induced by EPO. The cells were stained with Dylight 488 (red, representative of BrdU) and nuclear specific dye DAPI (blue). **E.** Cell number and BrdU content of 786-O and Caki-2 cells induced by EPO. All data are expressed as mean ± SEM. Each experiment was carried out in triplicates and the results of three independent experiments were used for statistical analysis. *p < 0.05, **p < 0.01 and ***p < 0.001 compared with the EPO untreated group (control).

On the basis of the bioinformatics analysis, Erythropoietin signaling pathway seemed to be engaged in ccRCC malignancy, which intrigued the interest of our further investigation. To examine whether ccRCC cells produce EPO, we measured EPO expression by ELISA. Quantification results showed that both 786-O and Caki-2 cells secreted EPO (from 14.35 to 41.35 pg/mL, Figure 3A), whereas the EPO secretion was 89% higher in 786-O compared to Caki-2 cells. To determine whether malignant RCC cells express EPO receptor (EPOR), 786-O and Caki-2 cells were lysed and examined. Figure 3B showed the Western Blot results of EPOR obtained from 786-O and Caki-2 cells, respectively. Lane 1 (786-O) and 2 (Caki-2) depict the immunoreactive EPOR protein bands, with an approximate molecular mass of 70 KD.

### Exogenous EPO promotes 786-O and Caki-2 cells proliferation

To examine the consequences of EPO exposure on ccRCC cells, we firstly treated 786-O and Caki-2 cells with a range of concentrations of exogenous r-Hu EPO (from 10 to 50 IU/mL) for 48 h and measured the relative cell viability using MTS assay. In the presence of 50 IU/mL r-Hu EPO, the proliferative ability of 786-O and Caki-2 cells were perceptibly enhanced compared to the vehicle group, suggesting r-Hu EPO has a stimulative effect on RCC cell proliferation (Figure 3C).

To further confirm the undesired pro-proliferative effect of r-Hu EPO-induced cell survival, multiparameter fluorescent high content screening (HCS) measurement was conducted. Simultaneous quantifications of multiparameter obtained from the same microscopic areas indicated that 50 IU/mL EPO dramatically increases tumor cell counts (BrdU, Figure 3D and 3E) and DNA content (DAPI, 2N verse 4N, Figure 3E), which illustrates that r-Hu EPO promotes 786-O and Caki-2 cells proliferative activity.

### Exogenous EPO increases migratory capacity in 786-O and Caki-2 cells

**Figure 4:**
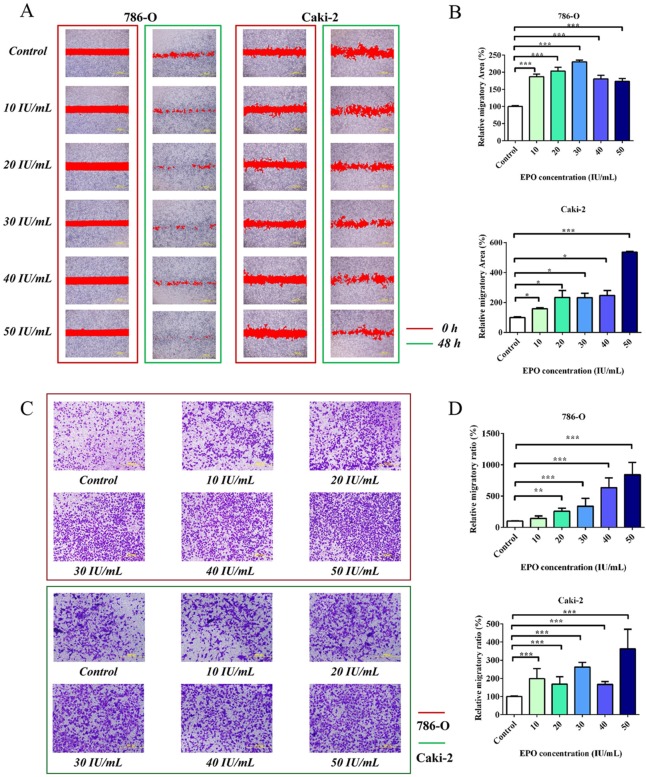
Migration assays of 786-O and Caki-2 cells induced by exogenous EPO **A.** The migratory ability of 786-O and Caki-2 cells induced by exogenous EPO was evaluated by the scratch assay; **B.** Statistical results of the migratory area induced by exogenous EPO in the scratch assay. **C.** Transwell results of 786-O and Caki-2 cells induced by exogenous EPO; **D.** Statistical results of the migratory ratio induced by exogenous EPO in the Boyden chamber assay. All data are expressed as mean ± SEM. Each experiment was carried out in triplicates and the results of three independent experiments were used for statistical analysis. *p < 0.05, **p < 0.01 and ***p < 0.001 compared with the EPO untreated group (control).

To evaluate the pro-metastatic ability of EPO on RCC *in vitro*, 786-O and Caki-2 cells were pre-cultured with serum free medium for 24 h and treated with r-Hu EPO (10 to 50 IU/mL). The wounding healing assay showed that both 786-O and Caki-2 cells had a faster migratory rate than the untreated cells among all of the concentrations used and 50 IU/mL EPO exhibited the strongest pro-migratory ability (Figure 4A and 4B). To further confirm the results mentioned above, we performed cell migration assay using the Boyden chamber. Consistent with the scratch assay, 786-O and Caki-2 cells treated with EPO displayed significant more migratory ability than the control group, resulting in more tumor cells across the Transwell membrane (Figure 4C and 4D). Thus, these results suggested that r-Hu EPO could accelerate the proliferative and migratory ability in 786-O and Caki-2 cells.

### Identification of high confidence predicted protein targets induced by exogenous r-Hu EPO in 786-O cell

**Figure 5:**
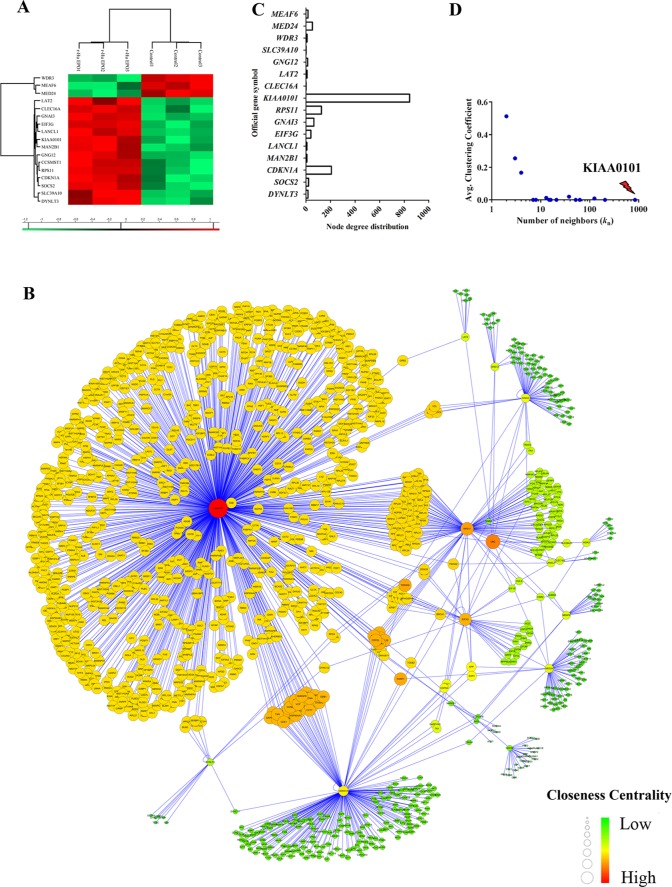
Construction of ccRCC regulatory network induced by exogenous EPO **A.** Heatmap visualization of the significantly expressed protein in 786-O cells treated with 50 IU/mL exogenous r-Hu EPO; **B.** Network model of human renal cell carcinoma proliferation and migration induced by exogenous EPO. The nodes (circle) in the networks represented protein, while the edge represented connections among nodes. **C.** Node degree distribution and **D.** the average clustering coefficient (*CC*) of the root proteins in the regulatory networks induced by exogenous EPO.

To understand how r-Hu EPO regulates RCC proliferation and migration, we introduce a proteomics profiling in quiescent 786-O cell with or without EPO treatment. Analysis of the control and EPO treated 786-O cell protein fractions reveals a high degree of overlap among each biological replication. Of the 4,781 proteins identified by LC-LTQ-Orbitrap-MS (Supplementary material 2), only 17 proteins were identified to be differently expressed in the EPO treated 786-O cell compared to the control groups (Table 2 and Figure 5A).

**Table 2:** Summary of differently expressed proteins in r-Hu EPO-treated 786-O cells

Prot ID	Prot full name	Gene name	*t*-test Difference	Regulated pattern
P51808	Dynein light chain Tctex-type 3	*DYNLT3*	−1.63242	up
B4DDR8	Mediator of RNA polymerase II transcription subunit 24	*MED24*	0.628352	down
F8VS53	Suppressor of cytokine signaling 2	*SOCS2*	−1.66598	up
P38936	Cyclin-dependent kinase inhibitor 1	*CDKN1A*	−2.84139	up
O00754–2	Lysosomal alpha-mannosidase	*MAN2B1*	−0.33492	up
O43813	LanC-like protein 1	*LANCL1*	−0.173473	up
O75821	Eukaryotic translation initiation factor 3 subunit G	*EIF3G*	−0.472412	up
P08754	Guanine nucleotide-binding protein G(k) subunit alpha	*GNAI3*	−0.302302	up
P62280	40S ribosomal protein S11	*RPS11*	−1.27588	up
Q15004	PCNA-associated factor	*KIAA0101*	−1.82851	up
Q2KHT3–2	Protein CLEC16A	*CLEC16A*	−0.904458	up
Q4G0I0	Protein CCSMST1	*CCSMST1*	−0.825412	up
Q9GZY6	Linker for activation of T-cells family member 2	*LAT2*	−0.371642	up
Q9HAF1–2	Chromatin modification-related protein MEAF6	*MEAF6*	2.86912	down
Q9UBI6	Guanine nucleotide-binding protein G(I)/G(S)/G(O) subunit gamma-12	*GNG12*	−1.15031	up
Q9ULF5	Zinc transporter ZIP10	*SLC39A10*	−1.50252	up
Q9UNX4	WD repeat-containing protein 3	*WDR3*	1.39392	down

In order to construct a reliable PPI network mediated by r-Hu EPO, 17 proteins differently expressed in the one-Dimensional SDS-PAGE proteomics assay were imported into the integration package of BisoGenet. As shown in Figure 5B, the PPI network mediated by r-Hu EPO contained 17 root proteins and 1,242 interactors. That is, all together, 1,259 nodes and 1,426 interactions. To identify and pinpoint the proteins that are most likely to drive the aggressive phenotype of RCC induced by EPO, we calculated the topological parameters. For each seed proteins, we counted the number of neighbors in the regulatory network and average clustering coefficient (*CC)* distribution. As shown in Figure 5C and 5D, among these root nodes regulated by EPO, KIAA0101 was ranked as the top roots and interacted with 843 transcription factors with the lowest average *CC* parameter (*CC*_KIAA0101_=0).

### KIAA0101 protein is associated with ccRCC proliferation and migration induced by r-HuEPO

**Figure 6:**
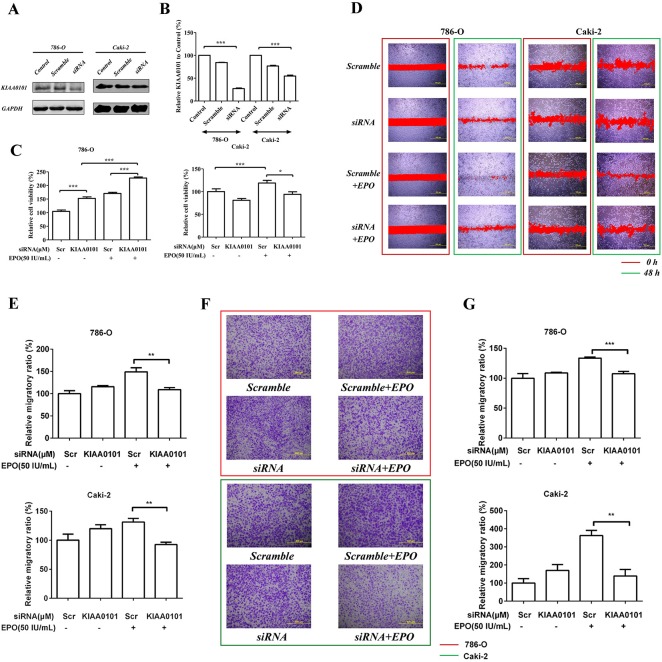
Cell proliferation and migration induced by r-Hu EPO before and after KIAA0101 knockdown **A.** siRNA knockdown and **B.** knockdown efficiency of KIAA0101 protein in 786-O and Caki-2 cells. **C.** Cell viability of 786-O and Caki-2 cell transfected with or without KIAA0101. Scramble (Scr) siRNA was used as positive control. **D.** Scratch analysis of exogenous EPO on 786-O and Caki-2 cells transfected with or without KIAA0101. **E.** Statistical results of the migratory ratio induced by EPO on 786-O and Caki-2 cells transfected with or without KIAA0101 in the scratch assay. **F.** The migration of exogenous EPO on 786-O and Caki-2 cells transfected with or without KIAA0101 using Transwell assay; **G.** Statistical results of the Transwell assay induced by EPO on 786-O and Caki-2 cells transfected with or without KIAA0101 in the Boyden chamber assay. All data are expressed as mean ± SEM. Each experiment was carried out in triplicates and the results of three independent experiments were used for statistical analysis. *p < 0.05, **p < 0.01 and ***p < 0.001 compared with the vehicle group.

In the PPI network, we found many transcription factors such as PDXK (pyridoxal (pyridoxine, vitamin B6) kinase), NOP58 (NOP58 ribonucleoprotein) and RPL7A (ribosomal protein L7a) were directly interacted with KIAA0101 and an imbalance of these regulations would result in aberrant nuclear-transcribed mRNA catabolic processes. Thus, to test the cause-effect of KIAA0101 protein expression, we knocked down KIAA0101 in 786-O and Caki-2 cells using RNA interference (Figure 6A and 6B). For the proliferation assay, MTS results showed that EPO treatment caused marked cell viability loss with a significant reduction of 25% in si-KIAA0101 Caki-2 cells. However, as to the 786-O cell, knockdown of KIAA0101 could not antagonize the pro-proliferative capacity by EPO (Figure 6C). As expected in wound healing (Figure 6D and 6E) and Transwell assays (Figure 6F and 6G), down-regulation of KIAA0101 in 786-O and Caki-2 cells attenuated their metastatic capacity induced by r-Hu EPO. These results indicated that KIAA0101 knockdown decreased ccRCC cells migration in both of these two ccRCC cells, and antagonized cell growth in Caki-2 cells.

### Exogenous EPO increases KIAA0101 protein expression

**Figure 7:**
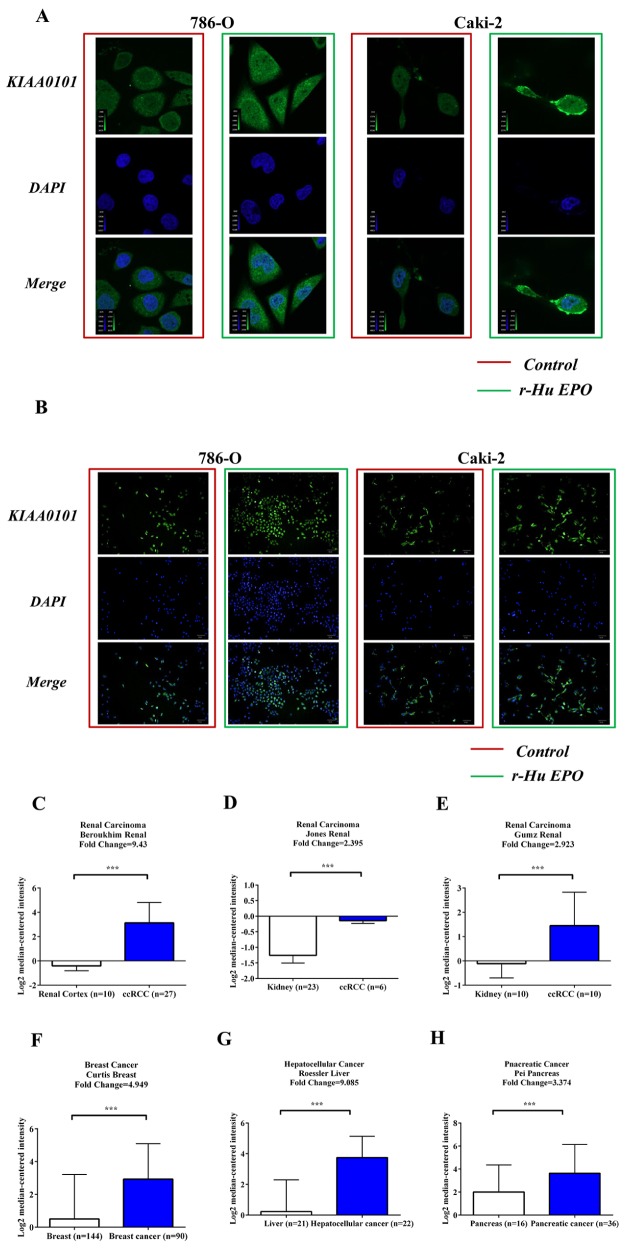
EPO promotes KIAA0101 expression in 786-O and Caki-2 cells and levels of KIAA0101 expression in normal tissues and primary tumors **A.** Protein levels of KIAA0101 with or without EPO treatment in 786-O and Caki-2 cell by Confocal analysis. **B.** Protein levels of KIAA0101 with or without EPO treatment in 786-O and Caki-2 cell by HCS. **C–H.** mRNA levels of *KIAA0101* in normal tissue and primary tumor of renal (C-E), breast (F), liver (G) and pancreas (H) Expression levels are presented as boxplots using median ± upper/lower limits, and were compared using an unpaired Student's *t* test. *p < 0.05, **p < 0.01 and ***p < 0.001 compared with the normal tissue groups.

Expression of KIAA0101 immunoreactivity with or without EPO treatment was conducted using HCS and confocal microscopy assay. Figure 7A indicated that KIAA0101 fluorescent intensity was dramatically up-regulated in ccRCC cells when exposed to 50 IU/mL r-Hu EPO. These data are also in consistent with the observations of HCS, as shown in Figure 7B. Thus, r-Hu EPO could enhance ccRCC cells malignancy *via* up-regulation the level of KIAA0101 protein.

### *KIAA0101* and *EPO* expression negatively correlate with 5-year survival in cancer patients

To investigate the clinical significance of *KIAA0101* in cancer, firstly, we searched the Oncomine database (https://www.oncomine.org) for *KIAA0101* mRNA expression between normal tissues and primary tumors. As shown in Figure 7C to 7E, mRNA levels of *KIAA0101* were significantly up-regulated in the primary tumor tissues of patients with renal carcinoma in many independent studies. Similarly, *KIAA0101* was also found to be elevated in multiple cancer subtypes, such as breast (Figure 7F), hepatocellular (Figure 7G) and pancreatic cancers (Figure 7H).

**Figure 8:**
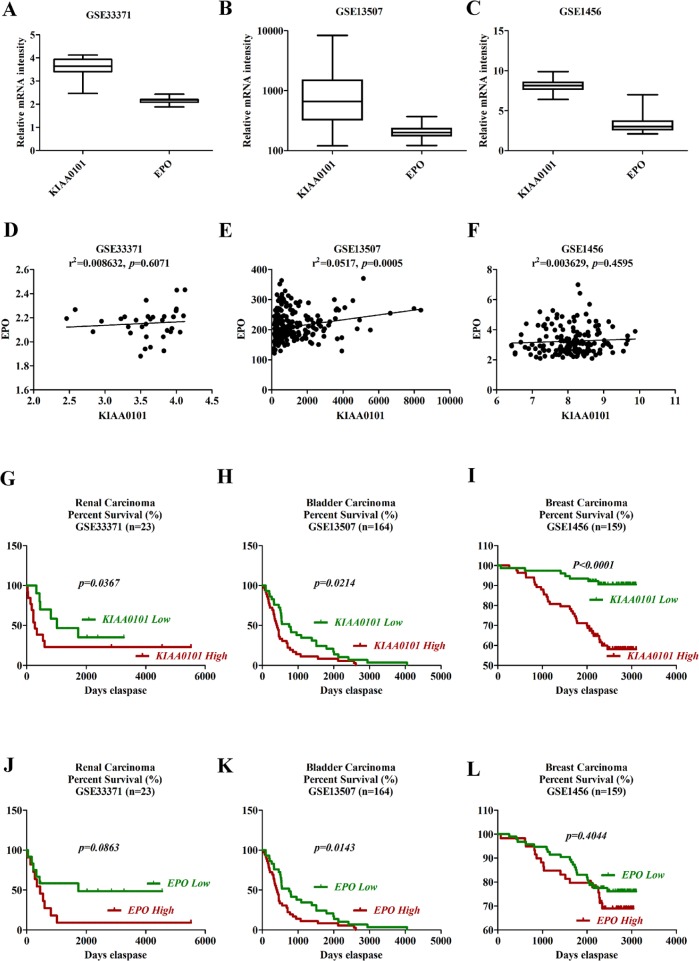
Survival analysis of *KIAA0101* and *EPO* in clinical cancer samples **A–C.** Boxplots of mRNA levels of *KIAA0101* and *EPO* in renal (A), bladder (B) and breast cancers (C) **D–F.** Correlation analysis of *KIAA0101* and *EPO* expression in clinical renal (D), bladder (E) and breast (F) cancer tissues. **G–I.** Kaplan-Meier survival analysis of *KIAA0101* in renal (G), bladder (H) and breast cancers (I) Overall survival was compared between high and low *KIAA0101* expression group using a median method as the bifurcating gene expression default setting. **J–L.** Kaplan-Meier survival analysis of *EPO* in renal (J), bladder (K) and breast cancers (L) Overall survival was compared between high and low *EPO* expression group using a median method as the bifurcating gene expression default setting. *p < 0.05, **p < 0.01 and ***p < 0.001 for Log-rank (Mantel-Cox) Test.

For the prognostic value of *KIAA0101* and *EPO* expression in the primary tumors, GSE33371 (renal carcinoma), GSE13507 (bladder carcinoma) and GSE1456 (breast carcinoma) were downloaded from Gene Expression Omnibus (GEO) database. As shown in Figure 8A to 8C, the mRNA levels of *KIAA0101* were much higher than *EPO* in cancer samples. In addition, *KIAA0101* and *EPO* expression were found to be positively correlated in the dataset of 23 renal carcinoma, 164 bladder carcinoma and 159 breast carcinoma samples (Figure 8D to 8F). As to renal carcinoma, patients with low levels of *KIAA0101* and *EPO* had a relative higher 3 and 5-year survival rate (Figure 8G and 8J, *KIAA0101*: *p* = 0.0367, *EPO*: *p* = 0.0863). Similarly, the GSE13507 dataset also suggested levels of *KIAA0101* and *EPO* expression were negatively associated with the overall survival rate in bladder carcinoma patients (Figure 8H and 8K, *p* < 0.05). Besides, high levels of *KIAA0101* and *EPO* gene expression in cancer tissues from patients with breast cancer (GSE1456) revealed a shortened relapse-free survival rate, as indicated by Figure 8I and 8L. Thus, these finding mentioned above confirmed the negative clinical significance for *KIAA0101* and *EPO* in cancer patients, which spurred us on to greater efforts to further understand the nature of *KIAA0101* and *EPO* in tumorigenesis.

## DISCUSSION

Most cancer patients with RCC show various degree of anemia, a major source of morbidity and mortality with a poor life quality. Thus, it is therefore critical to better understand whether the correction of anemia in RCC patients would discriminate aggressive from indolent RCC. In this study, we demonstrated that human ccRCC cell lines expressed authentic EPOR and secreted endogenous EPO. The activation of Erythropoietin signaling pathway by r-Hu EPO accelerated ccRCC cell growth and metastasis *in vitro*. These results further proved the potential undesired side effects of EPO to treat or prevent cancer-associated anemia in clinical findings.

Our results and other previous publications supported that KIAA0101 protein is a negative regulator of cancer progression. Zhu *et al*. found protein levels of KIAA0101 were up-regulated in 61 human primary cancer tissues from patients with gastric cancer. Suppression of KIAA0101 by RNA interference inhibited cell viability and prolonged overall survival [18]. Besides, over-expression of KIAA0101 also predicts a poor prognosis in lung cancer patients [21]. Consistent with the previously published data, we discovered EPO increased KIAA0101 expression and inhibition of KIAA0101 using RNA interference attenuated cell growth and migration in 786-O and Caki-2 cells induced by EPO. To further determine if *KIAA0101* and *EPO* expression are of clinical relevance, we interrogated databases of gene expression in patient samples from GEO database. Multiple independent datasets of patient samples indicated that mRNA levels of *KIAA0101* were much higher in the primary tumor of patients with renal, breast, liver and pancreatic cancer. Clinical survival analysis also revealed that low levels of *KIAA0101* are associated with an increased longer survival time. Similarly, endogenous *EPO* expression was also negatively correlated with cancer patients' survival. In addition, correlated analysis indicated that the expression of *KIAA0101* and *EPO* expression was positively in clinical tumor samples. Therefore, EPO coordinates the expression of KIAA0101 that cooperate to accelerate ccRCC progression and decreased 3 and 5 year survival.

Unlike the Caki-2 cells, 786-O cell line lacks the *VHL* tumor suppressor gene [22]. Although the pro-migratory capacity of EPO were similar in both 786-O and Caki-2 cells, the mitogenic responses towards EPO were quite different before and after KIAA0101 knockdown. In 786-O cells, when knockdown of KIAA0101protein, r-Hu EPO still elicited its deteriousness on cell viability. In contrast, Caki-2 cells responded with decrease in proliferation when treated with 50 IU/mL EPO. The differences in growth response between 786-O and Caki-2 cells are quite puzzling and lack explanations. In our present study, we observed the increased proliferative advantage of *VHL*-deficient 786-O cells revealed a more aggressive phenotype compared with the Caki-2 cells. According to the previous publications, it is conceivable that loss of wild type VHL protein expression resulted in the enhancement of proliferative and angiogenic activity in patients with RCC, as described by Paulsen *et al*. [23]. Thus, these differences indicated the fact that only patients with *VHL* gene can be treated with EPO for RCC-induced anemia when co-administration with KIAA0101 inhibitors.

In conclusion, using an integrative systems biological approach, we determined the role of EPO signaling pathway in ccRCC progression, and identified an undesired role of KIAA0101 in ccRCC proliferation and migration induced by EPO, which have not previously been linked KIAA0101 to kidney cancer cachexia elicited by EPO.

## MATERIALS AND METHODS

### Microarray selection and metadata normalization

The raw human microarray transcription tissue expression profiles were retrieved and downloaded from GEO (http://www.ncbi.nlm.nih.gov/geo/) and Array Express (http://www.ebi.ac.uk/arrayexpress/). Totally, three ccRCC microarray datasets from the cohorts of Lenburg *et al*. [24], Gumz *et al*. [25] and Cifola *et al*. [26], that included RCC samples and the matching normal tissue samples from the same patient, were downloaded. To avoid the variable biases from artifacts and non-biological conditions, raw fluorescence files were normalized using Robust Multichip Average (RMA) algorithm basing on R environment (http://www.r-project.org/). To remove the non-biological experiment variation across different microarray individuals, AILUN [27] platform comparison (http://ailun.stanford.edu/) and ArrayMining [28] cross-study integration (http://www.arraymining.net/) were achieved as our previously described [29].

### Datasets integration

As a major challenge for gene signatures identification, limited sample of clusters prevent the robustness of cancer biomarkers. Mutated tumor suppressor buried in the tumor genomes often led to genomic instability, which in turn resulted in genomic rearrangements or even chromosomal fragment mismatching [30]. To overcome the problem of low sample sizes in typical microarray studies, various cross-study normalization algorithms such as empirical Bayes (EB) [31], median rank scores (MRS) or quantile discretization (QD) [32], NorDi [33], Quantile discretization normalization (QDISC) [32], XPN [34] and Median Rank Score Normalization (MNORM) [32] were employed to decrease non-biological bias and variance. Since EB method has been applied widely to a large variety of microarray datasets analysis owing to its ability to robustly handle high-dimensional data when sample sizes are small, in this present study, we used EB algorithm to adjust for batch effects normalization for ccRCC [35].

### Microarray simulation and significantly expressed gene identification

High-throughput screening (HTS) technique facilitates rapid translation of molecular targets identification to clinical diagnostics and therapeutics. However, tumor heterogeneity is still a large challenge which prevents the robustness of gene signatures especially when the sample size was relative small [36]. In order to overcome such a disposition and provide robust cancer biomarkers, we carried out a simulated study from the integrated cohort *via* generating 8 virtual microarray data sets. For each virtual microarray data set, SAM [37] was applied for gene signatures identification. Gene expression was considered to be different if the threshold of false discovery rate (*FDR*) less than 0.05 and fold change above 1.2.

### RCC regulated network construction

Manually curated human signaling atlas with great superiority was downloaded from Edwin Wang lab (http://www.cancer-systemsbiology.org/) which covered data source from BioCarta, CST signaling pathways, pathways interaction database (PID), iHOP and even many review papers from cell signaling [38]. To obtain a related network regulated by renal cell carcinoma, differently expressed genes originated from the simulated microarray metadata were matched into the human cancer protein interaction network and visualized using Cytoscape (http://www.cytoscape.org/).

### Signaling pathway enrichment analysis by ingenuity pathway analysis

All the gene sets regulated by ccRCC were uploaded to the Ingenuity Pathway Analysis platform (IPA, Ingenuity System Inc, USA, http://www.ingenuity.com/) in the context of global canonical pathway. The *p* value was calculated using the right-tailed Fisher Exact Test.

### Cell culture

Two established human renal clear adenocarcinoma cell lines Caki-2 and 786-O were all purchased from ATCC (ATCC, Rockville, MD, USA). Cells were maintained in RPMI 1640 (786-O) or McCoy's 5A (Caki-2) medium supplemented with 10% (v/v) fetal bovine serum (FBS), 1% (v/v) penicillin and 1% (v/v) streptomycin. Cells were maintained at 37°C with 5% CO_2_ and proper humidity.

### MTS assay for cell proliferation

To elevate whether r-Hu EPO elicit a negative proliferative effect towards human ccRCC, cell viability was determined using MTS (CellTiter 96^®^ Aqueous, Madison, WI, USA) kit. Caki-2 and 786-O cells were seeded into the 96-well plates at a density of 2,000 per well and allowed to adhere using serum-free culture medium. After 24 h incubation, cells were treated with or without r-Hu EPO for another 48 h. At the end of incubation, 100 μL of MTS was added to each well and continued to incubate for 2 h at 37°C in a humidified atmosphere containing 5% CO_2_. Finally, to determine the amount of soluble formazan produced by cellular reduction of MTS, cells were recorded using a 96-well plate reader (Bio-Rad Laboratories Inc., Hercules, CA, USA) at 490 nm.

### High content screening (HCS) for BrdU proliferation

The Cellomics BrdU cell proliferation reagent kits (Thermo Scientific, Pittsburgh, PA, USA) was used for quantification of DNA replication per our previous publication [39]. As an alternative to ^3^H-thymidine, 5-bromo-2′-deoxyuridine (BrdU) enables us to detect DNA replication in actively proliferating cells using a monoclonal antibody against BrdU and DyLight 488 fluorophore-conjugated secondary antibody. Moreover, cell number and DNA content were quantified with DAPI staining. For image acquisition, Caki-2 and 786-O cells were fixed and analyzed using the high content screening Operetta system (Perkin-Elmer, Hamburg, Germany).

### Cell migration assays

To test the directional migratory ability of renal cell carcinoma cells before and after r-Hu EPO treatment, wound closure assay was performed. Briefly, confluent Caki-2 or 786-O cells were cultured in serum-depleted medium for 24 h and scratched with a 200 μL pipette tip and washed by germfree PBS three times. Wounds in the present or absent of r-Hu EPO were then imaged at different intervals with a Nikon Eclipse microscope (Nikon UK Limited, Kingston Upon Thames, UK).

For Boyden chamber assay, the bottom of the Transwell membrane was pretreated with Matrigel (Becton-Dickinson, Bedford, MA, USA) for 4 h. Afterwards, 5 × 10^4^ cells resuspended in serum-free r-Hu EPO medium were plated in the upper chamber of a transwell apparatus (8.0 μm pore, Coaster, Corning, NY, USA), while 600 μL of RPMI 1640 (786-O) or McCoy's 5A (Caki-2) medium with 10% FBS were provided in the lower chamber. After incubation at 37°C for 24 h, cells in the upper chamber were removed with a cotton sticker. Cells that migrated to the bottom of the membrane were attached and fixed, stained with 0.5% crystal violet and counted using Image Pro Plus software (Media Cybernetics, Inc., Bethesda, MD).

### Whole cell lysates and one-Dimensional SDS-PAGE separation

786-O Cells with or without r-Hu EPO treatment were washed with chilled PBS and lysed in RIPA buffer (50 mm Tric-HCl, (pH 7.5), 150 mm NaCl, 1 mm MgCl_2_, 1 mm CaCl_2_ and 1% Triton X-100) containing protease inhibitors (Roche Applied Science, Indianapolis, IN). The concentration of whole proteins was determined at 570 nm using the bicinchoninic acid kit (BCA, Pierce, Rockford, IL, USA). For one-Dimensional SDS-PAGE analysis, 80 μg of protein samples were separated using 12% SDS-PAGE gel. Gel was stained using coomassie blue (0.5% Coomassie Blue G-250, 30% methanol and 10% acetic acid) for 1 h and destained in 50% acetonitrile containing 5% acetic acid until the desired contrast was achieved.

### In-gel digestion, peptide extraction and separation, and liquid chromatography LTQ-orbitrap mass spectrometry (MS) analysis

Destained gel was cutted into 10 pieces and digested overnight with 5 μL trypsin (10 ng/μL) at 37°C. For peptide extraction, the trypsin digestions were extracted using filter aided sample preparation (FASP) per the manufacturer's protocol [40]. The extracted peptide samples were reconstituted with 600 μL of 20 mM ammonium formate at pH10.0, and directly injected into a C_18_ Waters XBridge BEH130 column (2.1 × 150 mm, Waters, Milford, MA) containing 3.5 μm particles. Peptides separation was performed in a Liquid Chromatography for gradient elution using acetonitrile plus 20 mM ammonium formate at a flow rate of 230 μL/min. As to the MS analysis, peptides were analyzed by a LTQ-OrbitrapVelos mass spectrometer (Thermo Fisher Scientific, Bremen, Germany) equipping with a Nanospray Flex Ion Source (Thermo Fisher Scientific, USA). Full scan MS spectra (m/z 350–2000) was monitored with a resolution of 60,000 at m/z 400. Finally, sequence analysis was carried on the Uniprot human protein database (http://www.uniprot.org/, release 3.43, 72,340 sequences) and database retrieval was performed on the Andromeda search engine based on probabilistic scoring [41].

### Raw data analysis and differentially expressed proteins identification

Relative protein abundance was calculated using MaxQuant [42] (http://www.maxquant.org/) and pre-processed using the filter function button in Perseus platform (version 1.5.2.6, http://www.perseus-framework.org/). For significantly expressed proteins identification, two class paired student's *t* test was utilized between the 3 r-Hu EPO treated groups and their matched vehicles with the *P* value less than 0.001.

### Protein-protein interaction network construction

To infer a reliable PPI network mediated by EPO in renal cell carcinoma, differently expressed protein identified by one-Dimensional SDS-PAGE proteomics were used as seeds to fish out the direct and indirect interactions from DIP (Database of Interaction Proteins, http://dip.doe-mbi.ucla.edu/dip/Main.cgi), BIOGRID (Biological General Repository for Interaction Datasets, http://thebiogrid.org/), HPRD (Human Protein Reference Database, http://www.hprd.org/), BOND (Bimolecular Object Network Database, http://bind.ca), MINT (Molecular Interaction database, http://mint.bio.uniroma2.it/mint/Welcome.do) and IntAct (http://www.ebi.ac.uk/intact/) warehouses from the in-house SysBiomics platform [43].

### Network optimization, topological calculation and target identification

For network optimization, the undirected PPI network was visualized using Cytoscape platform and Steiner minimal tree algorithm was applied for network optimization. By applying the NetworkAnalyzer plug- in [44], all directed and undirected self-loops were removed. In addition, nodes without connections were deleted from the network and only the largest component was considered as the renal cell carcinoma PPI network mediated by r-Hu EPO.

To calculate the regulatory capacity of each individual protein regulated by r-Hu EPO, again, the Cytoscape NetworkAnalyzer package was employed for topological parameters calculation. Degree (*k*_n_), the number of first links attached to a node that represents the capacity encoding by other transcription factors, was used and calculated in the network for neighborhood connectivity. In addition, we also quantify the structural properties of the renal cell carcinoma elicited by r-Hu EPO by its characteristic average clustering coefficient (*CC*) for affiliation networks, defined as followed:

$$\text{CC}=2e_{n}/(k_{n}(k_{n}-1))$$

Where *e_n_* represents the number of connected pairs between all neighbors of *n* [45].

### Western blot

Total protein extracted from Caki-2 and 786-O cells were lysed using RIPA buffer (Biyuntian, Shanghai, China) and equal amounts of proteins were separated using 12% SDS-PAGE electronics. Proteins were transferred to polyvinylidene difluoride (PVDF) membranes, blocked by 5% bovine serum albumin (BSA) and incubated with a rabbit monoclonal anti-KIAA010 antibody (diluted 1:1,000, Abcam, Cambridge, MA, USA), a rabbit monoclonal anti-EPOR antibody(diluted 1:2,000, Bioworld Technology, Inc. Minneapolis, MN) and a rabbit monoclonal anti-GAPDH antibody (diluted 1:10,000, Sigma-Aldrich, St Louis, MO, USA), followed be a DyLight 800-conjugated secondary immunoglobulin G antibody against rabbit (diluted 1:1,000, EarthOx, San Francisco, CA, USA).

### Immunofluorescence staining

Caki-2 and 786-O cells were allowed to adhere to WillCo-35-mm confocal dishes (WPI, Sarasota, FL, Invitrogen) with a glass bottom. 50 IU/mL r-Hu EPO was added to the dish and cells were incubated for 48 h at 37°C. Cells were washed with PBS and fixed with 4% pre-warmed formaldehyde for 20 to 30 min at room temperature, following by permeabilization with 0.1% Triton X-100. The permeabilized cells were stained with KIAA0101 (diluted 1:500, Abcam, Cambridge, MA, USA) and DyLight 488 (diluted 1:1,000, EarthOx, San Francisco, CA, USA) antibodies. For image acquisition, cellular fluorescence was scanned and monitored with an UltraVIEW VOX Confocal imaging system (PerkinElmer, Cambridge, UK).

### KIAA0101 protein knockdown

KIAA0101 small interfering RNA (siRNA) and the negative control siRNA (Scramble) were purchased from Santa Cruz Inc. (Santa Cruz Biotechnology, Santa Cruz, CA). For KIAA0101 protein transient knockdown, Caki-2 and 786-O cells were co-transfected KIAA0101 siRNA (or scramble siRNA) and siRNA Transfection Reagent for 12 h. After incubation with culture medium supplemented with 10% FBS for another 12 h, cells were lysed and the knockdown efficiency was assessed using Western Blot.

### Enzyme-linked immunosorbent assay (ELISA)

After 24, 48 and 72 h of incubation with serum-free medium, culture supernants from Caki-2 and 786-O cells were spinned down at 13,000 rpm for 10 min for the determination of the endogenous EPO secretion using a commercial available ELISA kit (Cloud-Clone Corp, Houston, TX) per the manufacturer's instructions.

### KIAA0101 expression and survival analysis in clinical samples

Expression of *KIAA0101* in normal and cancer samples of kidney, breast, pancreas and liver were obtained from Oncomine database (https://www.oncomine.org/). The prognostic values of *KIAA0101* in the primary tumor of patients with renal, breast cancer and bladder carcinoma were assessed using the datasets downloaded from GEO database. Overall survival was compared between high and low *KIAA0101* expression group using a median method as the bifurcating gene expression default setting in SPSS version 18.0 (SPSS Inc., Chicago, IL, USA).

### Statistical analysis

Statistical analysis of the microarray dataset was performed using SAM with the *FDR* ≤5% and fold change ≥1.2. For proteomics analysis, two class paired student's *t* test was employed with *P* value <0.001. Each experiment was repeated at least three times and date was expressed as mean± standard error of measurement (SEM) using GraphPad Prism 6.0 (GraphPad Software, San Diego, CA) package.

## SUPPLEMENTAL TABLES







## Abbreviations

BIOGRID: Biological General Repository for Interaction Datasets
BOND: Bimolecular Object Network Database
BrdU: 5-bromo-2′-deoxyuridine
BSA: bovine serum albumin
CASS: cancer-associated systemic diseases
ccRCC: clear cell renal cell carcinoma
CC: clustering coefficient
DIP: Database of Interaction Proteins
ELISA: Enzyme-linked immunosorbent assay
EPO: erythropoietin
ESAs: erythropoiesis-stimulating agents
FASP: filter aided sample preparation
FBS: fetal bovine serum
FDR: false discovery rate
GEO: Gene Expression Omnibus
HCS: high content screening
HTS: high-throughput screening
HPRD: Human Protein Reference Database
IPA: Ingenuity Pathway Analysis
MINT: Molecular Interaction database
MS: mass spectrometry
PID: pathways interaction database
PPI: protein-protein interaction
PCA: principal component analysis
PVDF: polyvinylidene difluoride
r-Hu EPO: recombinant human erythropoietin
RMA: Robust Multichip Average
SEM: standard error of measurement
SAM: Significance Analysis of Microarray
siRNA: small interfering RNA
TCGA-KIRC: the cancer genome atlas-kidney clear cell carcinoma
VHL: von Hippel-Lindau.
