# Supplementary material for: KIAA0101 is associated with human renal cell carcinoma proliferation and migration induced by erythropoietin
Source: Oncotarget. 2015 Nov 12;7(12):13520–37. doi: 10.18632/oncotarget.5876 (PMC4924658; doi:10.18632/oncotarget.5876)
Supplement: Supplementary file 1 [file oncotarget-07-13520-s001.pdf]

SUPPLEMENTARY TABLES

Supplementary Table S1: The integrated microarray dataset of ccRCC originated from three public available microarray datasets using EB algorithm

Supplementary Table S2: Proteomics expression profiling of relative protein abundances elicited by 50 IU/mL r-Hu EPO in 786-O cells
